# Transcription Factor MaHMG, the High-Mobility Group Protein, Is Implicated in Conidiation Pattern Shift and Stress Tolerance in *Metarhizium acridum*

**DOI:** 10.3390/jof11090628

**Published:** 2025-08-27

**Authors:** Rongrong Qiu, Jinyuan Zhou, Tingting Cao, Yuxian Xia, Guoxiong Peng

**Affiliations:** 1 Genetic Engineering Research Center, School of Life Sciences, Chongqing University, Chongqing 401331, China; qrr2629075405@163.com (R.Q.); 18338567490@163.com (J.Z.); c2276949015@163.com (T.C.); 2 Chongqing Engineering Research Center for Fungal Insecticide, Chongqing 401331, China; 3 Key Laboratory of Gene Function and Regulation Technologies Under Chongqing Municipal Education Commission, Chongqing 401331, China

**Keywords:** entomopathogenic fungi, *MaHMG*, gene knockout, conidiation, environmental tolerance

## Abstract

Conidiation and stress tolerance are pivotal traits in entomopathogenic fungi, critically influencing their production costs and environmental tolerance. While the transcription factor high-mobility group protein (HMG), characterized by a conserved HMG-box domain, has been extensively studied for its role in sexual development, its functions in entomopathogenic fungi remain largely unexplored. This study employed gene knockout to investigate the role of MaHMG in *Metarhizium acridum*. The deletion of *MaHMG* delayed conidiation initiation and caused a highly significant 58% reduction in conidial yield versus that of the wild type (WT) after 15 days. Furthermore, the conidiation pattern on microcycle induction medium (SYA) shifted from microcycle to normal conidiation. The Δ*MaHMG* mutant exhibited decreased conidial germination rates and markedly reduced tolerance following UV-B irradiation and heat-shock treatments, alongside increased sensitivity to the cell wall perturbant calcofluor white (CFW). RNA-seq analysis during this conidiation shift identified 88 differentially expressed genes (DEGs), with functional annotation implicating their predominant association with hyphal development, cell wall biogenesis, cell cycle progression, and conidiation. In conclusion, MaHMG functions as a critical positive regulator governing both conidiation and stress tolerance in *M. acridum*, underscoring its fundamental role in fungal biology and potential as a target for enhancing biocontrol agent performance.

## 1. Introduction

Entomopathogenic fungi constitute predominant insect pathogens and serve as critical natural regulators of insect population dynamics, being extensively deployed globally for managing diverse crop pests [1,2]. As the primary reproductive and dispersal units of these fungi, conidia determine key biocontrol characteristics; notably, conidial yield and stress tolerance significantly influence production costs and field utilization efficacy [3]. Filamentous fungi exhibit distinct conidiation patterns classified as normal sporulation or microcycle sporulation, with the latter conferring advantages in pathogenic species, including accelerated conidiation rates, uniform conidial morphology, and enhanced quality [4,5,6]. Consequently, elucidating the mechanistic basis of microcycle conidiation induction has emerged as a priority research focus in insecticidal mycology.

Normal conidiation represents the dominant reproductive strategy of filamentous fungi in natural environments, mediated by conidiophore development and governed by an intricate regulatory network [7]. Within this network, the core pathway comprising BrlA, AbaA, and WetA orchestrates conidiation-specific gene expression, determining sequential gene activation during conidiogenesis and conidial maturation. This pathway is activated by six upstream transcriptional activators (FluG, FlbA-E) and balanced by the repressor SfgA [8,9]. Microcycle conidiation functions as a stress adaptation strategy, enabling rapid proliferation through repetitive conidiation in response to nutrient limitation, salinity extremes, or thermal stress. Research on the regulation of conidiation pattern shifts in *M. acridum* has identified multiple genes modulating microcycle conidiation, including *MaCts1* [10], *MaEng1* [11], and the transcription factors *MaNCP1* [12] and *MaPac2* [13]. Deletion of these genes shifts conidiation from microcycle to normal patterns on SYA medium. These findings demonstrate a multifaceted regulatory landscape governing conidiation plasticity, though the precise molecular pathway coordinating this transition remains incompletely elucidated.

Entomopathogenic fungi, as living microorganisms, face diverse environmental stressors in nature—including UV-B radiation, elevated temperatures, and chemical agents—that contribute to inconsistent field efficacy [14]. UV-B exposure induces DNA damage within fungal conidial nuclei [15], primarily countered through two repair pathways: photoreactivation and nucleotide excision repair (NER). In photoreactivation, the white collar protein (WC) regulates UV tolerance mediators by modulating genes encoding DNA repair enzymes [16]; photolyase Phr is indispensable for light-dependent DNA repair [17]; and DNA endonuclease Uve1 initiates the UV damage response by excising lesions such as cyclobutane pyrimidine dimers (CPDs) and 6-4 photoproducts (6-4 PPs) [18]. Concurrently, the Rad gene family encodes core NER pathway components essential for UV stress resistance in these fungi [19]. High temperatures inflict damage upon DNA, proteins, and cell membranes; disrupt cell cycle progression and macromolecular synthesis; and may ultimately trigger cell death [20]. Induced under thermal stress, fungal heat shock proteins (HSPs) assist in proper protein folding, restoring native conformation and biological activity to play a pivotal role in thermotolerance [21]. Similarly, trehalose, renowned for its chemical stability, enhances abiotic stress resilience by stabilizing proteins and cellular membranes. The cell wall itself functions as a dynamic, plastic barrier against physicochemical environmental factors, regulating permeability while protecting against mechanical and osmotic stress [22]. This protective role is exemplified by the effector protein Tge1 from *Metarhizium*, which binds insect β-glucan receptors GNBP3 and GL3 to impede recognition of fungal cell wall components and suppress Toll pathway activation [23]. Elucidating novel stress tolerance-associated genes will deepen our understanding of resistance mechanisms in entomopathogenic fungi and advance strategies for targeted strain improvement.

High-mobility group (HMG) proteins constitute a class of transcription factors ubiquitous in animals, plants, and fungi, characterized by a conserved 70–80 amino acid HMG-box domain. This domain confers high sequence-specific DNA-binding capacity, enabling regulation of diverse physiological functions [24]. Proteins harboring the HMG-box domain are implicated in sexual development and mating processes in filamentous fungi. Genes encoding HMG-box proteins exhibit significant evolutionary conservation, having been identified across diverse lineages, from vertebrates and poriferans to angiosperms, algae, and fungi [25]. The functional roles of HMG transcription factors have been elucidated in several fungal systems: in *Coprinopsis cinerea*, *pcc1* regulates fruiting body morphogenesis [26]; in *Schizosaccharomyces pombe*, Ste11 acts as a master regulator of sexual differentiation [27]; and in *Candida albicans*, Rfg1 serves as a context-dependent positive or negative regulator of filamentous growth [28]. A homolog of the HMG transcription factor, designated MaHMG, has been identified in *M. acridum*; however, its biological function remains unknown. Here, we employed *M. acridum* to investigate the impact of *MaHMG* on conidiation pattern shift and stress tolerance. Our results demonstrate that MaHMG functions as a key positive regulator of conidiation and stress adaptability in *M. acridum*. This finding provides crucial support for elucidating the regulatory network underlying microcycle conidiation and establishes a theoretical foundation for targeted strain improvement.

## 2. Materials and Methods

### 2.1. Strains and Growth Conditions

*M. acridum* strain CQMa102, preserved in the China General Microbiological Culture Collection Center (Accession No. CGMCC 0877) and molecularly confirmed with GenBank accession number GCA_000187405.1, was used in this study. Unless otherwise specified, all of the fungal strains used in this study were cultured on 1/4-strength Sabouraud dextrose agar containing yeast extract (1/4 SDAY; 1% dextrose, 0.25% mycological peptone, 0.5% yeast extract, and 2% agar, *w/v*) at 28 °C for 15 days. *M. acridum* strain CQMa102 exhibits normal sporulation on 1/4 SDAY medium.

### 2.2. Constructions of Mutants

The *MaHMG* (Gene ID: MAC_07229) deletion mutant was constructed by homologous recombination [13]. Briefly, the upstream (about 1.7 kb) and downstream fragments (about 2.1 kb) of the *MaHMG* coding sequence were amplified from the wild-type (WT) genome by PCR, and these two fragments were inserted into the pK2-PB vector harboring a bar cassette. The constructed pK2-PB-*MaHMG*-LR vector was transformed into *M. acridum* using the *Agrobacterium tumefaciens*-mediated method [29]. To construct the complementation strain (CP), the bar gene in pK2-PB was replaced in situ with a sur cassette, and then the ORF and upstream sequence of *MaHMG* were inserted into the plasmid. Quantitative reverse transcription PCR (qRT-PCR) was adopted to further verify the *MaHMG* disruption and complementation strains. The above-mentioned primers are recorded in Table S1.

### 2.3. Conidial Capacity Assays

To determine conidial yield, we aseptically prepared a conidial suspension at a concentration of 1 × 10^6^ conidia/mL containing 0.05% Tween-80, which was then filtered through four layers of sterile lens paper to remove hyphae. The conidial concentration was determined using a hemocytometer. For yield measurement, 2 μL of the conidial suspension (1 × 10^6^ conidia/mL) was inoculated into each well of a 12-well plate containing 2 mL of 1/4 SDAY, followed by incubation at 28 °C. Conidia were collected at 3, 6, 9, 12, and 15 days of incubation by dispersing the fungal samples in 0.1% Tween 80 (1 mL per well) with vortexing and quantified using a hemocytometer.

### 2.4. Observation of Conidiation Patterns and Hyphal Septum Assays

To observe the conidiation process of the fungal strains, we uniformly spread 100 μL of conidial suspension (1 × 10^7^ conidia/mL) on 1/4 SDAY plates and microcycle conidiation induction medium (SYA medium: 0.5% yeast extract, 3% sucrose, 0.05% MgSO_4_, 0.001% MnSO_4_, 0.05% KCl, 0.3% NaNO_3_, 0.1% KH_2_PO_4_, 0.001% FeSO_4_, and 2% agar, *w/v*). After incubation for 10, 12, 18, 24, and 36 h, approximately 1 cm^2^ of the culture medium was cut for microscopic observation. At 18, 24, and 36 h, hyphal samples were stained with 10 µL of CFW (50 µg/mL) for 2 min and examined under a fluorescence microscope. Finally, the apical cell lengths of mycelia were measured.

### 2.5. Stress Tolerance Assays

Fungal tolerance to UV-B irradiation and heat shock was assessed as described [30]. Briefly, 50 μL conidial suspension (1 × 10^7^ conidia/mL) was spread onto 1/4 SDAY plates. Plates were exposed to UV-B (1350 mW/m^2^) for 0.5, 1.0, 1.5, and 2.5 h. For heat shock, the suspension was incubated at 43 °C for 2, 4, 6, 8, and 10 h before spreading. All plates were incubated at 28 °C for 20 h, after which conidial germination rates were determined. Tolerance to hyperosmotic and oxidative stresses was assessed via spot assays on 1/4 SDAY plates supplemented with 1 M NaCl, 1 M sorbitol, or 6 mM H_2_O_2_, respectively. Fungal sensitivity to cell wall-perturbing agents was evaluated using spot assays on 1/4 SDAY plates amended with 0.01% *w/v* sodium dodecyl sulfate (SDS), 50 μg/mL calcofluor white (CFW), or 500 μg/mL Congo red (CR). The conidial suspension (2 μL of 1 × 10^6^ conidia/mL) from WT, Δ*MaHMG*, and CP strains was spotted onto plates with or without stressors and incubated at 28 °C for 6 days before colony imaging [3]. Colony diameters were measured, and the mean diameter of each group was calculated. Growth rate and relative growth inhibition (RGI) were determined using the following formulas:

Growth rate = (D_t_)/(D_ck_)

RGI = (D_t_ − D_ck_)/D_ck_

where

D_t_ = Mean colony diameter of treatment group;

D_ck_ = Mean colony diameter of the untreated control group.

### 2.6. Determination of Trehalose Content

The trehalose content was extracted as previously described [31]. In brief, 0.1 g conidia was homogenized with 1 mL extraction solution and ultrasonicated at 200 W (3 s pulse, 10 s interval, 30 cycles). The mixture was incubated at room temperature for 45 min with intermittent shaking, followed by centrifugation at 8000 rpm for 10 min to collect supernatant. Concurrently, the trehalose standard was diluted with distilled water to generate concentrations of 0, 3.125, 6.25, 12.5, 25, 50, and 100 μg/mL. For quantification, 0.25 mL of sample or standard was combined with 1 mL working solution, incubated at 95 °C for 10 min, and cooled to room temperature, and the absorbance was measured at 620 nm. Trehalose content was calculated using a standard curve.

### 2.7. RT-qPCR Assays

To determine target gene expression levels, total RNA was reverse-transcribed into cDNA using the PrimeScript™ RT Master Mix kit (TAKARA, Dalian, China). Gene-specific qPCR primers for target genes were designed against cDNA sequences using the NCBI Primer-BLAST online tool (https://www.ncbi.nlm.nih.gov/tools/primer-blast/ accessed on 26 April 2025), with *M. acridum* GAPDH primers (GAPDH-F/GAPDH-R) serving as internal controls. The 20 μL RT-qPCR reaction contained 1 μL cDNA template, 1 μL each of forward/reverse primers, 10 μL 2× SYBR Green Super mix, and 7 μL ddH_2_O. Following instrument preheating to 95 °C, amplification proceeded with optimized annealing/extension parameters. Standard curves and Ct values were generated from instrument data. Relative mRNA expression was calculated using the 2^−ΔΔCt^ method [32] based on Ct values and standard curves. All primers for RT-qPCR are shown in the Supplementary Material, Table S1.

### 2.8. RNA Sequencing

To reveal the mechanism underlying the *MaHMG* regulation of the conidiation pattern shift, RNA-seq was performed to identify the differentially expressed genes (DEGs) in WT versus ∆*MaHMG*. Samples of the WT and ∆*MaHMG* strains after 10 h of culture on SYA were collected for RNA extraction. Approximately 10 mg DNA-free RNA from each fungal sample was submitted to BGISEQ-500 (BGI, Shenzhen, China) in the Beijing Genomics Institution (Wuhan, China) with three biological replicates for each fungal strain. The DEGs were defined by a Q value ≤ 0.05 and |fold change| ≥ 1.0. The DEGs were annotated according to the NCBI protein databases. The RNA-seq data were uploaded to the NCBI BioProject database under the accession number PRJNA1128374.

### 2.9. Data Analysis

Using SPSS 26.0 software, we performed a differential significance analysis of different sample data. We used the T-test and Tukey’s LSD test to compare the difference in means between groups at a significance level of α = 0.05. In order to visually display the results of data analysis, we used GraphPad Prism 9 software to draw statistical graphs and Photoshop CS 2022 software to process statistical graphs. All experiments were repeated more than three times.

## 3. Results

### 3.1. Bioinformatics Analysis of MaHMG

**Figure 1:**
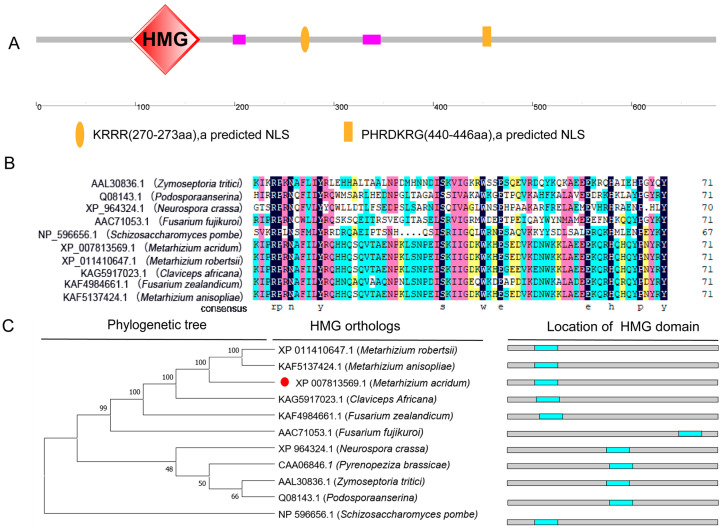
Structural and phylogenetic features of *MaHMG*. (**A**) Domain structure analysis of *MaHMG*. (**B**) Domain sequence alignments of *MaHMG* with other species. (**C**) Phylogenetic analysis of HMG protein sequence from different fungi and the location of HMG domain. Red circle represents HMG homologous protein in *M. acridum*.

The *MaHMG* gene has a coding sequence (CDS) of 2252 bp, containing a 194 bp intron, and encodes a 685-amino-acid protein (79.46 kDa). Prediction via the SMART online tool (http://smart.embl.de/ accessed on 8 June 2024) indicated that amino acid residues 95–165 contain an HMG-box domain. Nuclear localization signal (NLS) prediction using the online tool PSORT (http://psort.hgc.jp/form2.html/ accessed on 20 August 2024) identified two NLS motifs (residues 270–273 and 440–446), implying its potential role as a nuclear transcriptional regulator (Figure 1A). Multiple sequence alignment of HMG-box domains from various species conducted with DNAMAN version 8.0, showed high evolutionary conservation, and these domains exhibited high homology, indicating relatively conserved sequence features during evolution (Figure 1B). Phylogenetic analysis further demonstrated conserved evolution of HMG proteins in fungi, showing the closest evolutionary relatedness to other filamentous fungal species (Figure 1C).

**Figure 2:**
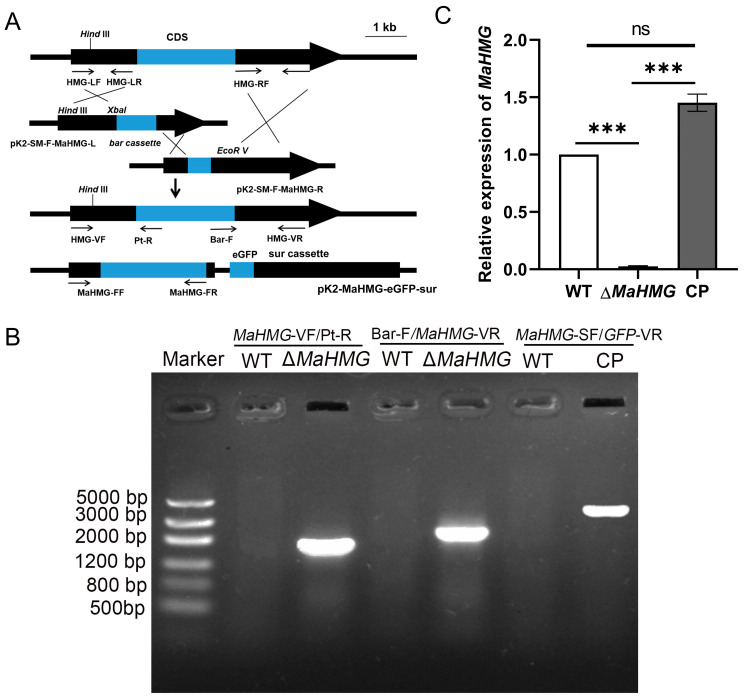
Construction of *MaHMG* knockout and complementary vectors and verification of the corresponding strains. (**A**) Schematic diagram of *MaHMG* knockout and complementary vector construction. The region between each pair of opposing arrows represents the gene fragment amplified using the corresponding forward and reverse primers. (**B**) PCR verification of knockout and complementary transformant. *MaHMG*-VF/Pt-R means to verify Δ*MaHMG* left arm (1765 bp); Bar-F/*MaHMG*-VR means to verify Δ*MaHMG* right arm (2179 bp); *MaHMG*-SF/GFP-VR means to verify reverting transformant (4409 bp). (**C**) Analysis of the *MaHMG* gene expression in each strain. The expression level of the *MaHMG* gene in WT was used as a control. WT: wild type; Δ*MaHMG*: *MaHMG* knockout strain; CP: complementary strain. ***: *p* < 0.001; ns: no significant difference. Error bars denote the standard deviation of the mean.

Based on homologous recombination and random insertion strategies, the *MaHMG* knockout vector (pK2-PB-bar-*MaHMG*) and complementation vector (pK2-PB-*MaHMG*-eGFP-Sur) were constructed (Figure 2A). The knockout strain (Δ*MaHMG*) and complemented strain (CP) were obtained via Agrobacterium-mediated transformation. Following PCR validation and RT-qPCR assessment, *MaHMG* transcript levels in Δ*MaHMG* were markedly reduced compared to those in WT and CP strains (*p* < 0.001; Figure 2B,C). These results confirmed the successful isolation of positive transformants.

### 3.2. MaHMG Deletion Reduces Conidiation Yield and Delays Conidiation in M. acridum

**Figure 3:**
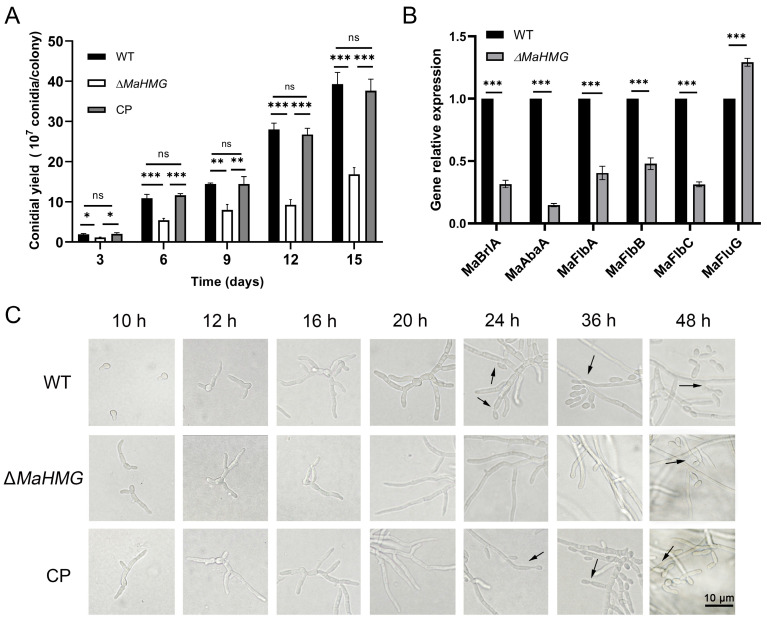
Conidiation of the fungal strains on 1/4 SDAY medium and quantification of gene expression related to the normal conidiation pathway. (**A**) Conidial yields of the WT, Δ*MaHMG*, and CP strains were assessed on 1/4 SDAY medium. (**B**) Quantification of gene expression related to the normal conidiation pathway. (**C**) Conidiation of the WT, Δ*MaHMG*, and CP strains on 1/4 SDAY medium, bar = 10 μm. (black arrow: normal conidiation). *: *p* < 0.05; **: *p* < 0.01; ***: *p* < 0.001; ns: no significant difference. Error bars denote the standard deviation of the mean.

To investigate the role of *MaHMG* in normal conidiation of *M. acridum*, conidiation yield and conidiation dynamics were assessed on 1/4 SDAY medium. Results showed that Δ*MaHMG* exhibited a highly significant reduction in conidiation yield compared to WT and CP strains (*p* < 0.001), with a 58% decrease relative to WT at day 15 (Figure 3A). Conidiation observation revealed that conidiophore structures appeared in WT and CP strains at 24 h, initiating conidiation. In contrast, conidiophores were not observed in Δ*MaHMG* until 48 h, demonstrating delayed conidiation onset and reduced conidiation in the mutant strain (Figure 3C).

RT-qPCR quantification of conidiation-related gene expression in Δ*MaHMG* revealed that transcript levels of *MaFlbA*, *MaFlbB*, *MaFlbC*, *MaBrlA*, and *MaAbaA* were significantly downregulated, whereas *MaFluG* expression was significantly upregulated (*p* < 0.001; Figure 3B). Homologs of these genes positively regulate conidiation in Aspergillus nidulans. This suggests *MaHMG* functions upstream of the *MaFlbs*-*MaBrlA*-*MaAbaA* module but downstream of *MaFluG*, positively regulating downstream genes while repressing *MaFluG* expression. Collectively, the delayed conidiation and reduced conidial yield in Δ*MaHMG* are likely mediated through dysregulation of key genes in the conidiation pathway.

### 3.3. MaHMG Deletion Shifts Conidiation Pattern from Microcycle to Normal Conidiation in M. acridum

**Figure 4:**
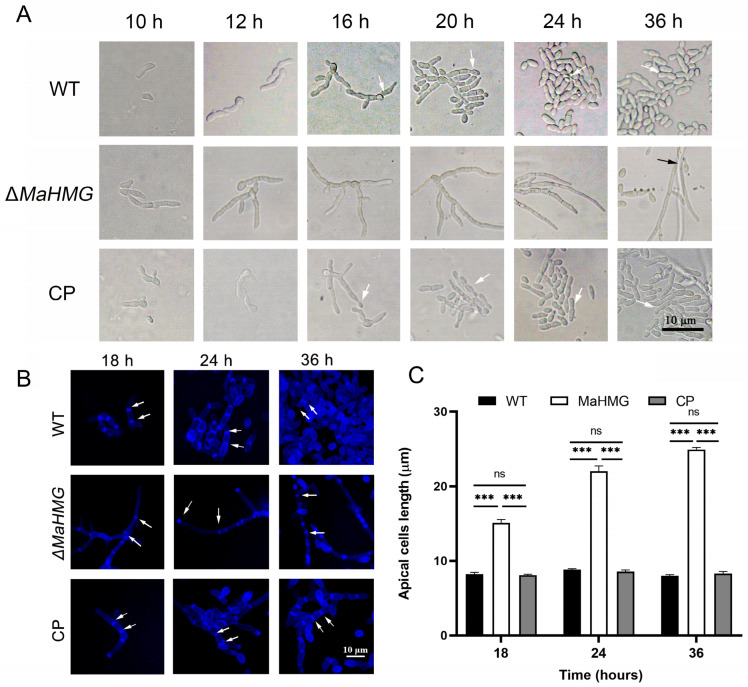
Conidiation of fungal strains on SYA medium. (**A**) Conidiation of the WT, Δ*MaHMG*, and CP strains on SYA medium, bar = 10 μm. (black arrow: normal conidiation; white arrow: microcycle conidiation). (**B**) Microscopic observation of chitin in hyphae stained with CFW. White arrows indicate hyphal septum interval; bar = 10 μm. (**C**) Length of hyphal apical cell of WT, Δ*MaHMG*, and CP strains. ***: *p* < 0.001; ns: no significant difference. Error bars denote the standard deviation of the mean.

Observation of conidiation dynamics on microcycle induction medium (SYA) revealed that WT and CP strains initiated microcycle conidiation at 16 h, whereas Δ*MaHMG* developed conidiophores and initiated conidiation at 36 h. This indicates that *MaHMG* deletion delays conidiation and shifts the conidiation pattern from microcycle to normal conidiation (Figure 4A).

After calcofluor white (CFW) staining, chitin in Δ*MaHMG* was not only localized at hyphal tips and septa but also displayed irregular distribution along extended hyphae compared to WT and CP strains (Figure 4B). Statistical analysis of inter-septal distances demonstrated that Δ*MaHMG* possessed significantly elongated hyphal compartments compared to WT (*p* < 0.001; Figure 4C), indicating that *MaHMG* likely regulates chitin biosynthesis. Its deletion causes disrupted chitin organization, hyphal over-elongation, and impaired conidiogenesis, ultimately leading to the conidiation pattern shift.

### 3.4. MaHMG Deletion Compromises UV-B and Heat-Shock Tolerance and Enhances Sensitivity to Cell-Wall-Perturbing Agents in M. acridum

**Figure 5:**
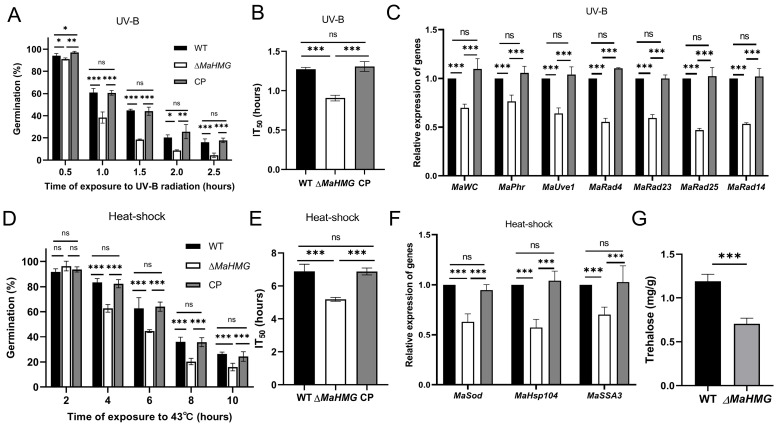
Assessment of UV-B irradiation and heat-shock tolerance. (**A**) Germination rates of the WT, Δ*MaHMG*, and CP strain conidia after 0.5, 1.0, 1.5, 2.0, and 2.5 h of UV-B irradiation (1350 mW/m^2^). (**B**) Median inhibition time (IT_50_) of the WT, Δ*MaHMG*, and CP strains after UV-B irradiation. (**C**) Expression of UV-B resistance-related genes in fungal strains after 1.0 h of UV-B irradiation. (**D**) Germination rates of the WT, Δ*MaHMG*, and CP strain conidia after heat shock at 43 °C for 2, 4, 6, 8, and 10 h. (**E**) Median inhibition time (IT_50_) of the WT, Δ*MaHMG*, and CP strains after heat shock. (**F**) Expression of heat-shock resistance-related genes in fungal strains after heat shock at 43 °C for 4 h. (**G**) Intracellular trehalose accumulation in 15-day-old aerial conidia incubated on 1/4 SDAY. *: *p* < 0.05; **: *p* < 0.01; ***: *p* < 0.001; ns: no significant difference. Error bars denote the standard deviation of the mean.

The germination rates of *M. acridum* conidia were evaluated following UV-B irradiation and heat shock for various durations. After 1.0 h of UV-B exposure, the germination rate of the Δ*MaHMG* strain was significantly reduced to 38.3 ± 4.11% compared to that of the WT strain (61 ± 1.70%) (*p* < 0.001; Figure 5A). The median inhibition time (IT_50_) for WT, Δ*MaHMG*, and CP were 1.27 ± 0.02, 0.90 ± 0.03, and 1.31 ± 0.05 h, respectively (Figure 5B). The UV-B IT_50_ of ΔMaHMG was 0.37 h shorter than that of WT. Consistently, key DNA repair genes—including photoreactivation components (*MaWC*, *MaPhr*, *MaUve1*) and NER pathway genes (*MaRad4*, *MaRad23*, *MaRad25*, *MaRad14*)—showed marked downregulation in Δ*MaHMG* (*p* < 0.001; Figure 5C).

After 4 h at 43 °C, the germination rate of Δ*MaHMG* was 62.67 ± 2.49%, significantly lower than that of WT at 83.33 ± 2.50% (*p* < 0.001; Figure 5D). The heat-shock IT50 of Δ*MaHMG* was 5.18 ± 0.09 h, approximately 1.71 h shorter than that of WT (6.89 ± 0.34 h) (Figure 5E). Moreover, heat-protective genes *MaSod*, *MaSSA3*, and *MaHsp104* were significantly downregulated in Δ*MaHMG* (*p* < 0.001; Figure 5F). Quantification of intracellular trehalose revealed a significant reduction in Δ*MaHMG*, which accumulated only 60% of the WT levels (*p* < 0.001; Figure 5G). This finding accounts for the increased heat-shock sensitivity of Δ*MaHMG*. Collectively, dysregulation of UV-repair genes, heat-protective genes, and trehalose biosynthesis in Δ*MaHMG* underlies the reduced UV-B and heat-shock tolerance in *M. acridum*.

**Figure 6:**
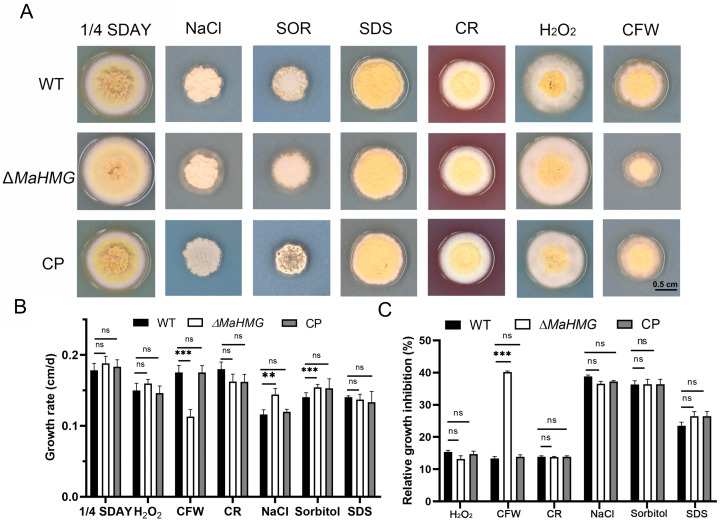
Chemical resistance analysis. (**A**) Colony growth status of each strain on various stress media supplemented with different chemical reagents, bar = 0.5 cm. (**B**) Growth rate of each strain on different stress media. (**C**) Relative growth inhibition rate (RGI) of the fungal strains. **: *p* < 0.01; ***: *p* < 0.001; ns: no significant difference. Error bars denote the standard deviation of the mean.

Under stress conditions, including hyperoxia, hyperosmolarity, and cell wall/membrane disruptants, the growth of the Δ*MaHMG* strain was examined. Δ*MaHMG* exhibited increased sensitivity to CFW, showing reduced colony size and significant growth inhibition. Additionally, on chemical-free 1/4 SDAY medium, Δ*MaHMG* formed slightly larger colonies with more densely branched hyphae, while on media supplemented with NaCl or SOR, it showed more vigorous hyphal growth (Figure 6A). Colony diameters across different media were measured to calculate growth rates and relative inhibition rates. Growth rates of Δ*MaHMG* were significantly reduced on CFW-containing medium (*p* < 0.001; Figure 6B), and relative inhibition rates were significantly higher than those of WT and CP strains (*p* < 0.001; Figure 6C). This indicates reduced CFW tolerance in Δ*MaHMG*, likely associated with compromised structure or function of the cell wall.

### 3.5. Identification of MaHMG-Regulated Differential Genes During Conidiation Pattern Shift by RNA-seq

**Figure 7:**
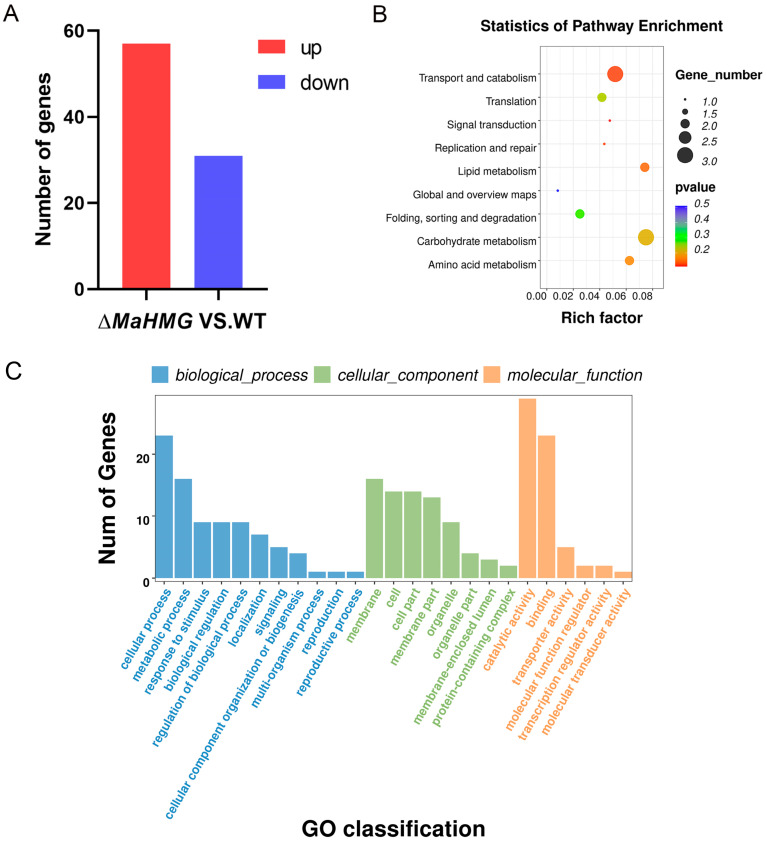
Number of DEGs in the conidiation stage transcriptome and analysis of the KEGG pathway and GO functional enrichment. (**A**) Number of upregulated and downregulated DEGs. (**B**) Enrichment analysis of DEGs in the KEGG pathway. (**C**) GO functional enrichment analysis of DEGs.

To investigate the molecular mechanisms of *MaHMG*-mediated conidiation pattern shift in *M. acridum*, transcriptome analysis was performed via RNA-seq using samples collected during conidiation on SYA medium. Differentially expressed genes (DEGs) were identified using the following thresholds: “log_2_(Δ*MaHMG*/WT) ≥ 1, Qvalue ≤ 0.05”. Analysis identified 57 significantly upregulated genes (17 encoding hypothetical proteins) and 31 downregulated genes (7 encoding hypothetical proteins) in Δ*MaHMG* versus WT (Figure 7A). To validate RNA-seq reliability, 20 randomly selected genes were subjected to qRT-PCR verification. Expression trends of 11 upregulated and 9 downregulated genes were fully consistent with RNA-seq results, confirming the reliability of our RNA-seq data (Figure S1).

Kyoto Encyclopedia of Genes and Genomes (KEGG) pathway enrichment analysis of DEGs using an enrichment bubble chart (Figure 7B) showed that DEGs were primarily enriched in pathways including transport and catabolism, translation, replication and repair, lipid metabolism, carbohydrate metabolism, and amino acid metabolism. Carbohydrate metabolism contained the highest number of enriched DEGs, while transport and catabolism exhibited the most significant enrichment. At the conidiation stage, *MaHMG* likely influences conidiation performance and pattern in *M. acridum* by modulating biochemical processes such as carbohydrate and amino acid metabolism, thereby affecting conidiation performance and patterning.

Gene Ontology (GO) enrichment analysis of DEGs revealed their distribution across 25 subcategories within three major domains: Biological Process, Cellular Component, and Molecular Function (Figure 7C). The Biological Process domain contained the most abundant terms, including cellular process, metabolic process, and response to stimulus, indicating that *MaHMG* modulates diverse biological processes during growth and conidiation in *M. acridum*. Published studies indicate cellular processes regulate fungal cell differentiation and development while also being crucial for cellular growth and proliferation [33]. Within Cellular Component, terms such as membrane and cell were enriched, where genes associated with membrane composition/structure influence conidial morphogenesis and hyphal development by modulating membrane biogenesis and architecture [34]. Molecular Function was predominantly enriched in catalytic activity and binding. Genes encoding catalytic functions are implicated in nutrient uptake and utilization during conidial development [35], while molecular binding factors govern conidial adhesion and cytokinesis, thereby modulating conidiation efficiency [36].

## 4. Discussion

RNA-seq profiling of WT and Δ*MaHMG* strains at 10 h post-inoculation on SYA medium revealed differentially expressed genes (DEGs) functionally annotated to hyphal morphogenesis, conidiation, and cell wall organization. Notably, MAC_02643 (a putative C6 zinc finger transcription factor), orthologous to pcz1 in Penicillium roqueforti, modulates vegetative growth, conidiogenesis, and conidial germination [37]. The MAC_01488-encoded NADPH oxidase, homologous to ClNOX2 in Curvularia lunata, enhances hyphal proliferation upon knockout, confers resistance to cell wall stressors, and accelerates developmental transitions, including conidial germination and appressorium differentiation [38]. Deletion of MAC_05411 (a subtilisin-like protease) in Botrytis cinerea severely impairs hyphal growth, sclerotia production, and conidiation [39]. MAC_06379 (a serine/threonine protein phosphatase) is essential for hyphal morphogenesis in Candida albicans [40]. MAC_04279 (a putative methionine permease) ortholog Met6 reduces mycelial pigmentation, aerial hyphae formation, and conidiation when deleted [41]. The MAC_07571-encoded putative cryptochrome DASH, orthologous to cryD in Fusarium fujikuroi, functions as a nitrogen-responsive transcriptional repressor of macroconidia production [42]. MAC_04339 (a GPI-anchored protein, putative) mediates cell wall reorganization, host adhesion, and biofilm development [43]. Collectively, these findings suggest that *MaHMG* likely coordinates the expression of these target genes to regulate development and conidiation patterning in *M. acridum*.

In *Aspergillus nidulans*, the *BrlA*→*AbaA*→*WetA* cascade constitutes the core conidiation regulatory pathway, which integrates environmental cues with developmental programs and is positively regulated by the Flbs complex (*FlbA*-*FlbD*) [7,44,45]. Upstream, *FluG* synthesizes diffusible signaling molecules (e.g., dehydroaustinol/dehydrodeoxyaurostinol, DLA) during nutrient depletion, which activate the Flbs complex by inhibiting the FadA-cAMP-PKA G-protein cascade, thereby inducing expression of the core regulator BrlA. BrlA initiates conidiophore development and activates *AbaA*, which regulates conidial chain elongation and differentiation, while WetA mediates conidial maturation and liberation [44]. In the Δ*MaHMG* strain, expression of *MaFlbA*, *MaFlbB*, *MaFlbC*, *MaBrlA*, and *MaAbaA* was downregulated, whereas *MaFluG* was upregulated. We propose that HMG acts as a novel master regulator of conidiation, modulating the *BrlA*→*AbaA*→*WetA* pathway via Flbs while feedback-repressing *FluG* when present, thereby attenuating *FluG*-mediated conidiation control to prevent overconidiation and maintain conidial yield homeostasis.

In filamentous fungi, elongated inter-septal distances and irregular chitin distribution significantly impact conidiation. Research demonstrates that increased inter-septal spacing impedes cytoplasmic streaming and diffusion of signaling molecules (e.g., cAMP), reducing conidial yield and delaying sporulation kinetics [46]. As a core structural component of the cell wall, disordered chitin deposition compromises the mechanical integrity of conidiating structures (e.g., conidiophores) and obstructs chitinase-mediated conidial liberation [47]. In the Δ*MaHMG* strain, chitin localization was disrupted, occurring not only aberrantly at hyphal tips and septa but also randomly along hyphae. This mutant exhibited significantly longer inter-septal distances compared to WT and CP strains, indicating that *MaHMG* modulates conidiation patterning by regulating chitin organization and septal spacing in the fungal cell wall.

UV-B radiation induces pyrimidine dimer formation in DNA, impeding replication and transcription [15]. Current studies demonstrate that UV endonucleases (UVE1, WC) and photolyase (Phr) serve as critical protective factors by repairing these dimers, thereby preventing UV-B damage to mitochondrial genomes [48]. In *Saccharomyces cerevisiae*, *Rad25* is essential for nucleotide excision repair (NER) following UV irradiation [49]. In *Metarhizium robertsii*, *RAD4* exhibits induced expression during UV-induced NER [50]. In the Δ*MaHMG* strain, UV-B treatment caused significant downregulation of DNA repair genes (*UVE1*, *WC*, *Phr*, *RAD4*, *RAD14*, *RAD23*, *RAD25*). This demonstrates that *MaHMG* modulates UV tolerance by regulating the expression of DNA repair genes. Furthermore, RNA-seq identified dysregulation of stress-responsive genes, including UV endonuclease *UVE1* (MAC_07337), which promotes UV adaptation; its deletion in *Cryptococcus neoformans* increases UV sensitivity [51], consolidating *MaHMG*’s function in conidial stress adaptation.

Heat-shock proteins (HSPs), induced under thermal stress, facilitate proper protein folding and restoration of native conformation, playing pivotal roles in fungal thermotolerance [21]. In *Fusarium pseudograminearum*, *FpHsp104* (ortholog of yeast *Hsp104*) is crucial for heat resistance [52]. In *Saccharomyces cerevisiae*, *SSD1* regulates Hsp104-dependent disaggregation of protein aggregates and contributes to cell wall remodeling, thereby enhancing heat adaptation [53]. Trehalose functions as a bioprotectant that maintains protein structural integrity under heat stress, inhibits heat-induced aggregation, and significantly bolsters thermotolerance in fungi [54]. In *Schizosaccharomyces pombe*, heat-shock stress elevates intracellular trehalose, increasing osmotic pressure and activating the cell wall integrity pathway [55]. Following heat-shock exposure, the Δ*MaHMG* strain exhibited downregulation of key heat-shock genes (*MaSod*, *MaSSA3*, *MaHsp104*), reduced trehalose accumulation, and diminished thermotolerance.

The fungal cell wall, a dynamic structure of heterogeneous composition, is essential for cellular viability, morphogenesis, and pathogenesis. This outermost barrier protects against physicochemical stressors, regulates permeability, and mitigates mechanical and osmotic pressures [56]. Chemical susceptibility assays revealed increased sensitivity of the *ΔMaHMG* strain to CFW, exhibiting restricted hyphal growth and reduced colony diameter on 1/4 SDAY amended with CFW compared to WT and CP controls. This phenotype aligns with studies demonstrating that CFW and other cell-wall-targeting compounds induce severe morphological defects by disrupting wall integrity [57].

## 5. Conclusions

In summary, the transcription factor MaHMG governs the main biocontrol traits of *M. acridum*, including conidiation capacity, conidiation pattern, and stress tolerance. Conidiation pattern shift involves hyphal development, cell wall biogenesis, cell cycle progression, and the conidiation process. Our findings not only reveal the fundamental functions of *MaHMG* in *M. acridum* but also elucidate the molecular mechanisms governing conidiation pattern shift and diminished stress tolerance. Therefore, this study establishes a critical theoretical foundation for screening entomopathogenic fungi and promoting the application of fungal insecticides.

## Data Availability

RNA-seq data were deposited in the NCBI BioProject database (accession No. PRJNA1128374).

## Supplementary Materials

The following supporting information can be downloaded at: https://www.mdpi.com/article/10.3390/jof11090628/s1. Figure S1. RT-qPCR validation of DEGs identified in RNA-seq analysis. Table S1. Primers used in the study. Table S2. The DEGs in conidiation of ∆*MaHMG* compared to WT.

## References

[1] Wang C., Wang S. (2017). Insect pathogenic fungi: Genomics, molecular interactions, and genetic improvements. Annu. Rev. Entomol..

[2] Ortiz-Urquiza A., Luo Z., Keyhani N.O. (2015). Improving mycoinsecticides for insect biological control. Appl. Microbiol. Biotechnol..

[3] Zhou J., Wang S., Xia Y., Peng G. (2024). Maazar, a Zn2Cys6/fungus-specific transcriptional factor, is involved in stress tolerance and conidiation pattern shift in *Metarhizium acridum*. J. Fungi.

[4] Zhang S., Peng G., Xia Y. (2010). Microcycle conidiation and the conidial properties in the entomopathogenic fungus *Metarhizium acridum* on agar medium. Biocontrol Sci. Technol..

[5] Li S., Myung K., Guse D., Donkin B., Proctor R.H., Grayburn W.S., Calvo A.M. (2006). Fvve1 regulates filamentous growth, the ratio of microconidia to macroconidia and cell wall formation in *Fusarium verticillioides*. Mol. Microbiol..

[6] Son H., Kim M.G., Min K., Lim J.Y., Choi G.J., Kim J.C., Chae S.K., Lee Y.W. (2014). Weta is required for conidiogenesis and conidium maturation in the ascomycete fungus *Fusarium graminearum*. Eukaryot. Cell.

[7] Etxebeste O., Garzia A., Espeso E.A., Ugalde U. (2010). *Aspergillus nidulans* asexual development: Making the most of cellular modules. Trends Microbiol..

[8] Park H.S., Yu J.H. (2012). Genetic control of asexual sporulation in filamentous fungi. Curr. Opin. Microbiol..

[9] Seo J.A., Guan Y., Yu J.H. (2006). Flug-dependent asexual development in *Aspergillus nidulans* occurs via derepression. Genetics.

[10] Zou Y., Li C., Wang S., Xia Y., Jin K. (2022). Macts1, an endochitinase, is involved in conidial germination, conidial yield, stress tolerances and microcycle conidiation in *Metarhizium acridum*. Biology.

[11] Dai H., Zou Y., Xia Y., Jin K. (2024). Maeng1, an endo-1,3-glucanase, contributes to the conidiation pattern shift through changing the cell wall structure in *Metarhizium acridum*. J. Invertebr. Pathol..

[12] Li C., Xia Y., Jin K. (2022). The c2h2 zinc finger protein mancp1 contributes to conidiation through governing the nitrate assimilation pathway in the entomopathogenic fungus *Metarhizium acridum*. J. Fungi.

[13] Hu X., Li B., Li Y., Xia Y., Jin K. (2025). Mapac2, a transcriptional regulator, is involved in conidiation, stress tolerances and pathogenicity in *Metarhizium acridum*. J. Fungi.

[14] Tong S.M., Feng M.G. (2020). Phenotypic and molecular insights into heat tolerance of formulated cells as active ingredients of fungal insecticides. Appl. Microbiol. Biotechnol..

[15] Sancar A. (2003). Structure and function of DNA photolyase and cryptochrome blue-light photoreceptors. Chem. Rev..

[16] Idnurm A., Verma S., Corrochano L.M. (2010). A glimpse into the basis of vision in the kingdom Mycota. Fungal Genet. Biol..

[17] Shimura M., Ito Y., Ishii C., Yajima H., Linden H., Harashima T., Yasui A., Inoue H. (1999). Characterization of a *Neurospora crassa* photolyase-deficient mutant generated by repeat induced point mutation of the phr gene. Fungal Genet. Biol..

[18] Tagua V.G., Pausch M., Eckel M., Gutiérrez G., Miralles-Durán A., Sanz C., Eslava A.P., Pokorny R., Corrochano L.M., Batschauer A. (2015). Fungal cryptochrome with DNA repair activity reveals an early stage in cryptochrome evolution. Proc. Natl. Acad. Sci. USA.

[19] Chatterjee N., Walker G.C. (2017). Mechanisms of DNA damage, repair, and mutagenesis. Environ. Mol. Mutagen..

[20] Roti Roti J.L. (2008). Cellular responses to hyperthermia (40–46 °C): Cell killing and molecular events. Int. J. Hyperth..

[21] Sottile M.L., Nadin S.B. (2018). Heat shock proteins and DNA repair mechanisms: An updated overview. Cell Stress Chaperones.

[22] Lima S.L., Colombo A.L., de Almeida J.J.N. (2019). Fungal cell wall: Emerging antifungals and drug resistance. Front. Microbiol..

[23] Lu M., Wei D., Shang J., Li S., Song S., Luo Y., Tang G., Wang C. (2024). Suppression of *Drosophila* antifungal immunity by a parasite effector via blocking gnbp3 and gnbp-like 3, the dual receptors for β-glucans. Cell Rep..

[24] Malarkey C.S., Churchill M.E.A. (2012). The high mobility group box: The ultimate utility player of a cell. Trends Biochem. Sci..

[25] Foster J.W., Brennan F.E., Hampikian G.K., Goodfellow P.N., Sinclair A.H., Lovell-Badge R., Selwood L., Renfree M.B., Cooper D.W., Graves J.A. (1992). Evolution of sex determination and the Y chromosome: Sry-related sequences in marsupials. Nature.

[26] Murata Y., Fujii M., Zolan M.E., Kamada T. (1998). Molecular analysis of pcc1, a gene that leads to a-regulated sexual morphogenesis in *Coprinus cinereus*. Genetics.

[27] Sugimoto A., Iino Y., Maeda T., Watanabe Y., Yamamoto M. (1991). *Schizosaccharomyces pombe* ste11^+^ encodes a transcription factor with an hmg motif that is a critical regulator of sexual development. Genes Dev..

[28] Kadosh D., Johnson A.D. (2001). Rfg1, a protein related to the *Saccharomyces cerevisiae* hypoxic regulator rox1, controls filamentous growth and virulence in *Candida albicans*. Mol. Cell. Biol..

[29] Lazo G.R., Stein P.A., Ludwig R.A. (1991). A DNA transformation-competent Arabidopsis genomic library in *Agrobacterium*. Biotechnology.

[30] Ma Q., Jin K., Peng G., Xia Y. (2015). An ena ATPase, maena1, of *Metarhizium acridum* influences the Na^+^-, thermo- and UV-tolerances of conidia and is involved in multiple mechanisms of stress tolerance. Fungal Genet. Biol..

[31] Al-Naama M., Ewaze J.O., Green B.J., Scott J.A. (2009). Trehalose accumulation in *Baudoinia compniacensis* following abiotic stress. Int. Biodeterior. Biodegrad..

[32] Livak K.J., Schmittgen T.D. (2001). Analysis of relative gene expression data using real-time quantitative PCR and the 2^−ΔΔCT^ Method. Methods.

[33] Ribeiro L.F.C., Chelius C.L., Harris S.D., Marten M.R. (2017). Insights regarding fungal phosphoproteomic analysis. Fungal Genet. Biol..

[34] Roberts S.K., Milnes J., Caddick M. (2011). Characterisation of anbest1, a functional anion channel in the plasma membrane of the filamentous fungus, *Aspergillus nidulans*. Fungal Genet. Biol..

[35] Wang S., Lin R., Tumukunde E., Zeng W., Bao Q., Wang S., Wang Y. (2022). Glutamine synthetase contributes to the regulation of growth, conidiation, sclerotia development, and resistance to oxidative stress in the fungus *Aspergillus flavus*. Toxins.

[36] Peñalva M.A., Tilburn J., Bignell E., Arst H.N. (2008). Ambient ph gene regulation in fungi: Making connections. Trends Microbiol..

[37] Gil-Durán C., Rojas-Aedo J.F., Medina E., Vaca I., García-Rico R.O., Villagrán S., Levicán G., Chávez R. (2015). The pcz1 gene, which encodes a Zn(II)_2_Cys_6_ protein, is involved in the control of growth, conidiation, and conidial germination in the filamentous fungus *Penicillium roqueforti*. PLoS ONE.

[38] Wang F., Gao W., Sun J., Mao X., Liu K., Xu J., Fu D., Yuan M., Wang H., Chen N. (2020). NADPH oxidase clnox2 regulates melanin-mediated development and virulence in *Curvularia lunata*. Mol. Plant Microbe Interact..

[39] Liu X., Xie J., Fu Y., Jiang D., Chen T., Cheng J. (2020). The subtilisin-like protease bcser2 affects the sclerotial formation, conidiation and virulence of *Botrytis cinerea*. Int. J. Mol. Sci..

[40] Lee C.M., Nantel A., Jiang L., Whiteway M., Shen S.H. (2004). The serine/threonine protein phosphatase *SIT4* modulates yeast-to-hypha morphogenesis and virulence in *Candida albicans*. Mol. Microbiol..

[41] Saint-Macary M.E., Barbisan C., Gagey M.J., Frelin O., Beffa R., Lebrun M.H., Droux M. (2015). Methionine biosynthesis is essential for infection in the rice blast fungus *Magnaporthe oryzae*. PLoS ONE.

[42] Castrillo M., García-Martínez J., Avalos J. (2013). Light-dependent functions of the *Fusarium fujikuroi* cryd dash cryptochrome in development and secondary metabolism. Appl. Environ. Microbiol..

[43] Samalova M., Carr P., Bromley M., Blatzer M., Moya-Nilges M., Latgé J.P., Mouyna I. (2020). Gpi-anchored proteins in *aspergillus fumigatus* and cell wall morphogenesis. Curr. Top. Microbiol. Immunol..

[44] Adams T.H., Boylan M.T., Timberlake W.E. (1988). Brla is necessary and sufficient to direct conidiophore development in *Aspergillus nidulans*. Cell.

[45] Wieser J., Adams T.H. (1995). Flbd encodes a myb-like DNA-binding protein that coordinates initiation of *Aspergillus nidulans* conidiophore development. Genes Dev..

[46] Adams T.H., Wieser J.K., Yu J.H. (1998). Asexual sporulation in *Aspergillus nidulans*. Microbiol. Mol. Biol. Rev..

[47] Borgia P.T., Iartchouk N., Riggle P.J., Winter K.R., Koltin Y., Bulawa C.E. (1996). The chsb gene of *Aspergillus nidulans* is necessary for normal hyphal growth and development. Fungal Genet. Biol..

[48] Wong H.J., Mohamad-Fauzi N., Rizman-Idid M., Convey P., Alias S.A. (2019). Protective mechanisms and responses of micro-fungi towards ultraviolet-induced cellular damage. Polar Sci..

[49] Qiu H., Park E., Prakash L., Prakash S. (1993). The *Saccharomyces cerevisiae* DNA repair gene RAD25 is required for transcription by RNA polymerase II. Genes Dev..

[50] Zhang Y.L., Peng H., Zhang K., Ying S.H., Feng M.G. (2023). Divergent roles of Rad4 and Rad23 homologs in *Metarhizium robertsii*’s resistance to solar ultraviolet damage. Appl. Environ. Microbiol..

[51] Verma S., Idnurm A. (2013). The UVE1 endonuclease is regulated by the white collar complex to protect *Cryptococcus neoformans* from UV damage. PLoS Genet..

[52] Xia H., Chen L., Fan Z., Peng M., Zhao J., Chen W., Li H., Shi Y., Ding S., Li H. (2021). Heat stress tolerance gene *FpHsp104* affects conidiation and pathogenicity of *Fusarium pseudograminearum*. Front. Microbiol..

[53] Mir S.S., Fiedler D., Cashikar A.G. (2009). Ssd1 is required for thermotolerance and hsp104-mediated protein disaggregation in *Saccharomyces cerevisiae*. Mol. Cell. Biol..

[54] Argüelles J.C. (1997). Thermotolerance and trehalose accumulation induced by heat shock in yeast cells of *Candida albicans*. FEMS Microbiol. Lett..

[55] Péter M., Gudmann P., Kóta Z., Török Z., Vígh L., Glatz A., Balogh G. (2021). Lipids and trehalose actively cooperate in heat stress management of *Schizosaccharomyces pombe*. Int. J. Mol. Sci..

[56] Ibe C., Munro C.A. (2021). Fungal cell wall: An underexploited target for antifungal therapies. PLoS Pathog..

[57] Bermejo C., García R., Straede A., Rodríguez-Peña J.M., Nombela C., Heinisch J.J., Arroyo J. (2010). Characterization of sensor-specific stress response by transcriptional profiling of wsc1 and mid2 deletion strains and chimeric sensors in *Saccharomyces cerevisiae*. OMICS.

