# Supplementary material for: Transcription Factor MaHMG, the High-Mobility Group Protein, Is Implicated in Conidiation Pattern Shift and Stress Tolerance in Metarhizium acridum
Source: J Fungi (Basel). 2025 Aug 27;11(9):628. doi: 10.3390/jof11090628 (PMC12470670; doi:10.3390/jof11090628)
Supplement: Supplementary file 1 [file jof-11-00628-s001.zip › jof-3787132-supplementary.pdf]

# Transcription Factor MaHMG, the High-mobility group protein, Is implicated in Conidiation Pattern Shift and Stress Tolerance in *Metarhizium acridum*

--Supplementary Material

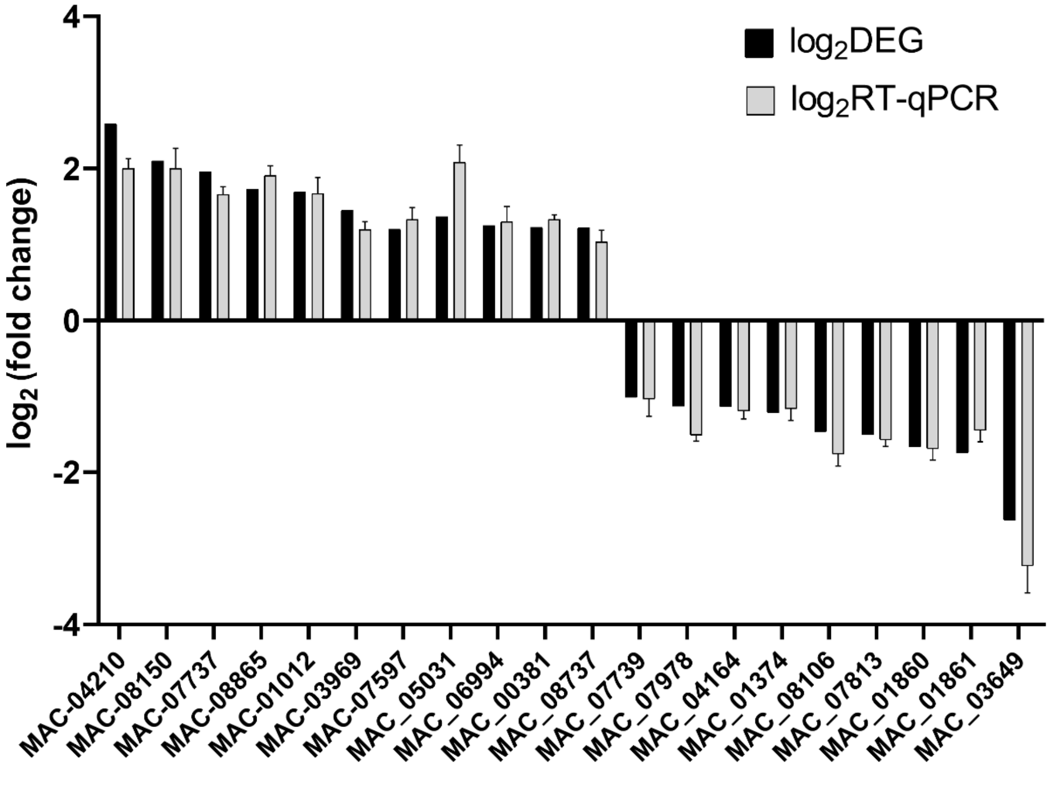


**Figure S1.** RT-qPCR validation of DEGs identified in RNA-seq analysis.

**Table S1.** primers used in the study.

| Primer | Sequence (5′-3′) | Description |
|---|---|---|
| *MaHMG* LF | ATAGGGCTTGATTTTGGCTT | Used to clone the 5′ end of *MaHMG* |
| *MaHMG* LR | GCACAGAATACCAGCGAATA | |
| *MaHMG* RF | ATTATGAAGAGAAACGAGGC | Used to clone the 3′ end of *MaHMG* |
| *MaHMG* RR | CGTCAATATCCTCTACAACA | |
| Bar-F | GCTCTACACCCACCTGCT | Used for screening the *MaHMG* disruption transformants |
| Pt-R | CAGCCAAGCCCAAAAAGTG | |
| *MaHMG* VF | CAACACAACAGCATTCTTGC | |
| *MaHMG* VR | AAACAAATGAAGGAGGCGGA | |
| *MaHMG* SF | GCTCTTTTTTTCTCCTTCAG | Used to form pK2- MaHMG-EGFP-SUR vector |
| *MaHMG* SR | AGGCGTTCTCCCGAGACTAA | |
| *MaHMG* QF | GGCACCCAAGATACCCAG | Used to analyze the expression of *MaHMG* by qRT-PCR |
| *MaHMG* QR | ATTGTGTTTGACGACTTCTGC | |
| GAPDH-QF | GACTGCCCGCATTGAGAAG | |
| GAPDH -QR | AGATGGAGGAGTGGGTGTTG | |
| 07377QF | GGGCACCTACGACAGGTATG | Used for the validation of gene transcription levels by RT-qPCR |
| 07377QR | AGGCGTAGAGGACCAGGAAT | |
| 09677QF | GAGCTTGGCTGTTCTTTGGC | |
| 09677QR | GAAGGCATACACCGAGGAGG | |
| 04712QF | CAAAACCAGGTGCAGATGCC | |
| 04712QR | CGATCGAGTACTGTCCAGGC | |
| 05417QF | AACCTTTGCCTCGGGTATGG | |
| 05417QR | GTGAGGGACTGTCCAACGAG | |
| 05625QF | TGTGGACTCCCCAGGTAGAG | |
| 05625QR | GAGAGTTGGGATGTGACGGG | |
| 07727QF | TTATGACGATGAGCCGACCG | |
| 07727QR | AGGCATCCAGTCCTGAGCTA | |
| 01374QF | GGTGGTGCTGGAATCAGTCA | |
| 01374QR | CGCGACACTCTCTCCAAACT | |
| 08106QF | TCGTTCGACTGAGCCATTCC | |
| 08106QR | TCTCCTATGGACAAGCCGGA | |
| 01860QF | TGTTGGACATGGGAATCGGG | |
| 01860QR | AAACTCCAGGCCGTACATGG | |

| | |
|---|---|
| 03649QF | TCCGGCCACGGTATTATTCG |
| 03649QR | GGCCCGAGTTGATTTCGTTG |
| 07337QF | CGAAGATGGCATTGACGCTG |
| 07337QR | GTTACATCCTGGCTGCCGTA |
| 08765QF | ACTTCACCAAGCCAGTCGAG |
| 08765QR | GGCTCTTGTTCAGTGCCTCT |
| 01012QF | GAAGAGCCGGACTGGATACG |
| 01012QR | TTGTCATGGCAGCAAAGCAC |
| 03969QF | ATTGCGTGGTTTGTTTCGGG |
| 03969QR | ATTGCTGGTTGGATGCTGGA |
| 07597QF | TACACCCTTTTGGGGATGGC |
| 07597QR | GCGGTCGCATTGTAATTCCC |
| 05168QF | CGGCAGTAAGGAAACTGGGT |
| 05168QR | ACCACGTGCTGTCAAGTAGG |
| 07037QF | TACGGCCCATGGAACAACAA |
| 07037QR | TAAGCGGGGTGTAGGTGACT |
| 06426QF | TGCCATCAAAAGACGAGCCT |
| 06426QR | GATGTAGGGCAGGGACGATG |
| 08417QF | TCACCTCAACCTCAACCACG |
| 08417QR | CTTGTCCTTGGCGTGGATCT |
| 02643QF | ATGTCCTCCCAAACACCGAC |
| 02643QR | GTGGGTGGACTTGGTGGTAG |
| *BrlA*-QF | GTGCTTTCGGGCTTCTTTGG |
| *BrlA*-QR | TGGGTTATCGTAGGTCCCGT |
| *AbaA*-QF | GCCAAGTTGCAGCATCACAA |
| *AbaA*-QR | CATCCACGGGAGACTCGATG |
| *FlbA*-QF | CCGACGAGGCGTACTACATC |
| *FlbA*-QR | CTCACTGCCTTTCCCCTCTG |
| *FlbB*-QF | ATGGCCCTCTACACTCCCTT |
| *FlbB*-QR | TCATGGTAGACGGCCCAGTA |
| *FlbC*-QF | TTACCCAATTCCTCCGCGAC |
| *FlbC*-QR | AGAGAGACCAGTCTCCCGTC |
| *FluG*-QF | CTTCTGTGCAAGAATCGCGG |
| *FluG*-QR | AAAGGTACAGGTCGTCGGTG |

| | |
|---|---|
| *RAD4*-QF | AAATGCGAAGGAAGCAGGGA |
| *RAD4*-QR | TCGAACGAGTTGCTGCAGAT |
| *RAD14*-QF | ACATGATGCTGTTCCTGCGA |
| *RAD14*-QR | TCTGTTCGAGTCTTGCGCTT |
| *RAD23*-QF | AGGAAGTGGTAGTGGCAACG |
| *RAD23*-QR | GAGCTGCTGAAACTGAGGGT |
| *RAD25*-QF | CAAGAGGTCACGCACTCCTT |
| *RAD25*-QR | CCACTCTTGGCTCGACCATT |
| *WC*-QF | TGTATCAACACTCCTTCAG |
| *WC*-QR | CGAGGTCAATAGCATCATA |
| *Phr*-QF | GAACAGACACTTGGAATC |
| *Phr*-QR | TGCCTAAGTAGAGATATGC |
| *UVE1*-QF | TAACAACGCACGAGATAT |
| *UVE1*-QR | AGGCTATAACCATACTCTG |
| *SOD*-QF | CGTTCCGACTATTGTATTACC |
| *SOD*-QR | ACAGGCTTGAGTATCAGTAG |
| *HSP104*-QF | AACCGTAATCTTGTCATC |
| *HSP104*-QR | AACCGTAATCTTGTCATC |
| *SSA3*-QF | ATTCTGCTGCTTGATGTT |
| *SSA3*-QR | TTCTTGGTGGGAATAGTG |

**Table S2.** Analysis of DEGs between the WT and *ΔMaHMG* strains, grown on SYA plates

| Gene ID | log2(Δ*MaHMG* /WT) | Up and Down regulation | Description |
|---|---|---|---|
| MAC_09523 | 3.459659373 | Up | hypothetical protein |
| MAC_04210 | 2.588105356 | Up | DNA mismatch repair protein, putative |
| MAC_01860 | 2.099518925 | Up | AAA family ATPase, putative |
| MAC_02662 | 1.997662199 | Up | hypothetical protein |
| MAC_07337 | 1.956019115 | Up | UV-endonuclease UVE-1 |
| MAC_03676 | 1.949480468 | Up | hypothetical protein |
| MAC_01666 | 1.919070475 | Up | prephenate dehydrogenase |
| MAC_00065 | 1.913480488 | Up | hypothetical protein |
| MAC_06395 | 1.906976398 | Up | activating signal cointegrator 1 complex subunit 3 |
| MAC_09579 | 1.790889978 | Up | hypothetical protein |
| MAC_08267 | 1.785544982 | Up | putative BCS1 protein precursor |
| MAC_05168 | 1.770425845 | Up | ZIP family zinc transporter |
| MAC_05122 | 1.736932026 | Up | hypothetical protein |
| MAC_03601 | 1.736155315 | Up | putative LTE1 protein |
| MAC_08765 | 1.727971823 | Up | pheromone-regulated multispanning membrane protein Prm1, putative |
| MAC_01497 | 1.701662332 | Up | hypothetical protein |
| MAC_02643 | 1.698191732 | Up | C6 finger domain-containing protein |
| MAC_01012 | 1.693996412 | Up | 6-phosphogluconate dehydrogenase, NAD-binding protein |
| MAC_07571 | 1.676736792 | Up | Putative cryptochrome DASH |
| MAC_09362 | 1.63107571 | Up | phosphate-repressible phosphate permease |
| MAC_08782 | 1.621359489 | Up | IQ calmodulin-binding motif protein |
| MAC_01488 | 1.619346674 | Up | NADPH oxidase |

| | | | |
|---|---|---|---|
| MAC_07735 | 1.613356713 | Up | hypothetical protein |
| MAC_08975 | 1.603807158 | Up | hypothetical protein |
| MAC_07037 | 1.599167797 | Up | protein MSF1 |
| MAC_05220 | 1.588687275 | Up | hypothetical protein |
| MAC_05764 | 1.57561779 | Up | TRAF-type zinc finger protein |
| MAC_04339 | 1.565670578 | Up | GPI anchored protein, putative |
| MAC_09665 | 1.55098986 | Up | kinesin |
| MAC_06426 | 1.55020748 | Up | TOR signaling pathway regulator (TapA) |
| MAC_03991 | 1.523554318 | Up | Mediator of RNA polymerase II transcription subunit 4 |
| MAC_06136 | 1.514486219 | Up | UPF0023 family protein |
| MAC_04487 | 1.505078571 | Up | hypothetical protein |
| MAC_03022 | 1.502948744 | Up | hypothetical protein |
| MAC_00052 | 1.481444488 | Up | mitochondrion organization and biogenesis protein |
| MAC_02010 | 1.457699994 | Up | hypothetical protein |
| MAC_03969 | 1.450122214 | Up | G-protein coupled receptor |
| MAC_05411 | 1.421798003 | Up | subtilisin-like protease |
| MAC_08516 | 1.420337734 | Up | GTPase activating protein (BUD2/CLA2) |
| MAC_08417 | 1.417660854 | Up | alpha/beta hydrolase, putative |
| MAC_05031 | 1.362717901 | Up | interferon-induced GTP-binding protein Mx |
| MAC_00278 | 1.33570383 | Up | hypothetical protein |
| MAC_03214 | 1.291031134 | Up | hypothetical protein |
| MAC_06994 | 1.248388748 | Up | interferon-induced GTP-binding protein Mx |
| MAC_00381 | 1.228060026 | Up | QDE-2-interacting protein |
| MAC_08737 | 1.218069571 | Up | GTPase activating protein Rga6 |
| MAC_06227 | 1.211892495 | Up | uracil DNA glycosylase superfamily protein |
| MAC_00970 | 1.209146154 | Up | hypothetical protein |
| MAC_07597 | 1.202817214 | Up | beta-lactamase, putative |

| | | | |
|---|---|---|---|
| MAC_07223 | 1.185097204 | Up | Pumilio-family RNA binding repeat protein |
| MAC_08908 | 1.144686896 | Up | glutaminyl-tRNA synthetase |
| MAC_02111 | 1.131358058 | Up | 66S ribosomal complex subunit |
| MAC_09554 | 1.09920465 | Up | aromatic amino acid aminotransferase |
| MAC_05771 | 1.062317657 | Up | serine/threonine protein kinase |
| MAC_04279 | 1.028382497 | Up | putative methionine permease |
| MAC_07160 | 1.026629274 | Up | hypothetical protein |
| MAC_03677 | 1.02251919 | Up | ATP-dependent RNA helicase DBP8 |
| MAC_07739 | -1.02109384 | Down | integral membrane protein |
| MAC_07978 | -1.131731079 | Down | L-ornithine 5-monooxygenase (L-ornithine N(5)-oxygenase) |
| MAC_04164 | -1.141577879 | Down | integral membrane protein |
| MAC_01374 | -1.220608465 | Down | plasma membrane channel protein (Aqy1), putative |
| MAC_05057 | -1.393474487 | Down | hypothetical protein |
| MAC_02941 | -1.401158944 | Down | trans-sialidase |
| MAC_07769 | -1.419895271 | Down | hypothetical protein |
| MAC_08106 | -1.471790261 | Down | alcohol dehydrogenase, putative |
| MAC_06266 | -1.478953934 | Down | glycosyl transferase, putative |
| MAC_01612 | -1.505189741 | Down | mucin, putative |
| MAC_07813 | -1.511827129 | Down | muramidase, putative |
| MAC_07377 | -1.543311808 | Down | MFS multidrug transporter, putative |
| MAC_03715 | -1.570826533 | Down | hypothetical protein |
| MAC_08541 | -1.620896217 | Down | hypothetical protein |
| MAC_09677 | -1.665568845 | Down | ferrichrome-type siderophore transporter |
| MAC_06871 | -1.6670696 | Down | ubiquitin thiolesterase |
| MAC_01860 | -1.6679764 | Down | long-chain-fatty-acid-CoA ligase, putative |

| | | | |
|---|---|---|---|
| MAC_06379 | -1.674137345 | Down | serine/threonine protein phosphatase, putative |
| MAC_04712 | -1.685496426 | Down | zinc metalloproteinase |
| MAC_00781 | -1.687336032 | Down | phosphatidyl synthase |
| MAC_01861 | -1.747249892 | Down | peptide synthetase |
| MAC_01827 | -1.753921781 | Down | meiotically up-regulated protein |
| MAC_05417 | -1.75856039 | Down | amidohydrolase family protein |
| MAC_05625 | -1.764330321 | Down | C6 transcription factor |
| MAC_00510 | -1.789974795 | Down | hypothetical protein |
| MAC_09621 | -1.805287399 | Down | pyruvate formate lyase activating enzyme |
| MAC_07727 | -1.813337461 | Down | C2H2 finger domain protein, putative |
| MAC_08045 | -1.951727124 | Down | hypothetical protein |
| MAC_03649 | -2.630247885 | Down | cell surface protein (Mas1) |
| MAC_09760 | -5.677325755 | Down | hypothetical protein |
